# Harnessing power of simulation training effectiveness with Kirkpatrick model in emergency surgical airway procedures

**DOI:** 10.1016/j.heliyon.2022.e10886

**Published:** 2022-10-06

**Authors:** Nam-Hung Chia, Victor Kai-Lam Cheung, Madeleine Lok-Yee Lam, Iris Wai-Kwan Cheung, Taurus Kwun-Yip Wong, Sze-Sze So, Eric Hang-Kwong So, George Wing-Yiu Ng

**Affiliations:** a Multi-Disciplinary Simulation & Skills Centre (MDSSC), Queen Elizabeth Hospital, Hong Kong; b Department of Surgery, Queen Elizabeth Hospital, Hong Kong; c Department of Neuroscience, Psychology and Behaviour, University of Leicester, UK; d Department of Anaesthesiology & Operation Theatre Services, Queen Elizabeth Hospital, Hong Kong; e Intensive Care Unit, Queen Elizabeth Hospital, Hong Kong

**Keywords:** Training effectiveness, Cricothyroidotomy, Kirkpatrick, Simulation, Healthcare professionals

## Abstract

**Objectives:**

Training effectiveness indicates how good a program has met pre-set training objectives or organizational goals for the best benefit of healthcare professionals and service users in the community. The study aimed to evaluate training effectiveness following implementation of new training curriculum of emergency surgical airway procedures (Cricothyroidotomy) organized by the Queen Elizabeth Hospital.

**Design:**

This training evaluation relied on observational descriptive study design. Timed task on Cricothyroidotomy procedures and standardized post-training questionnaire were applied to assess the first 3 levels of Kirkpatrick's model: (Level-1) Reaction by training satisfaction; (Level-2) Learning by acquisition of knowledge and skills assessment passing rate; (Level-3) Behavior by personal strengths.

**Setting:**

This program was operated in the Multi-Disciplinary Simulation and Skills Centre, a hospital-based high-fidelity simulation training center accredited by the Society for Simulation in Healthcare.

**Participants:**

The study recruited 80 trauma service providers, including 35 general surgeons, 15 emergency physicians, 10 anesthesiologists or intensivists, 6 neurosurgeons, 4 orthopedic surgeons, and 10 emergency nurses from five trauma centers under the Hospital Authority. All underwent the Advanced Trauma Life Support training in advance.

**Results:**

Compared with reference score from previous training sessions, the result of program using new training curriculum and simulator demonstrated significant training satisfaction of participants (Level-1), and high level of assertiveness, mental preparedness, self-efficacy, and internal locus of control and responsibility (p < .01, for all in Level-3). All participants (N = 80) completed entire Cricothyroidotomy procedure in 2 min without technical errors (Assessment passing rate = 100%) (Level-2).

**Conclusions:**

Under Kirkpatrick model, simulation training in Cricothyroidotomy procedure using new curriculum and simulators has been proven to be useful for healthcare professionals involved in trauma service management. The result suggests that application of a state-of-the-art training tools to advanced surgical skills training could improve training satisfaction, knowledge and skills acquisition, and personal strengths transferable to clinical practice.

**ACGME competencies:**

Practice Based Learning and Improvement.

## 1 Introduction

Training effectiveness relies on systematic process in training evaluation [1, 2, 3]. Foci of training evaluation are on reviewing whether participants can meet expected organizational goals or training objectives, such as reaction or satisfaction with the training, acquisition of knowledge and skills through learning, behavior (including but not limited to specific action, procedure, and some relatively subtle change in psychological constructs or attitude in real work setting following the training), translational effects on clinical performance and safety, and monetary or societal values to the community as well as the high management of an organization for resources prioritization decision [2, 3, 4]. Training curriculum with standardized and validated materials, to a large extent, drives a training program towards achieving expected learning outcomes throughout the design, implementation, and evaluation phases. In 1959, Kirkpatrick established the first widely adopted model in training effectiveness at 4 levels [4].

• (Level-1) Reaction, addressing subjective feeling or satisfaction of trainees.
• (Level-2) Learning, addressing knowledge and skills acquired.
• (Level-3) Behavior, addressing knowledge, skills and/or other personal attributes transferred.
• (Level-4) Results, addressing ultimate performance or measurable impact on the organization.

### 1.1 Significance of cricothyroidotomy training needs for healthcare professionals

Cricothyroidotomy, or surgical airway procedure, has long been used as an effective damage control procedure on preventing death of anoxia following airway obstruction, in particular trauma cases in hospitals as well as emergency maxillofacial and neck injury in battlefields [5, 6]. Under “Cannot Intubate Cannot Oxygenate” condition, maintaining airway and oxygenation by open surgical airway procedure on throat with a scalpel is the only option for lifesaving purpose [7, 8, 9, 10, 11]. Despite its importance as first-line approach in airway management, only small proportions of emergency physicians or surgeons worldwide have undergone formal training before applying it to injured patients [9, 12, 13]. Needs of developing a simulator for medical professionals to practice this “low frequency but high impact procedure” have been identified in military and hospital. However, the knowledge gap in choosing or validating simulators was large, not to mention how it affects training effectiveness nor how it brings positive impact on organization management [14, 15, 16].

**Figure 1:**
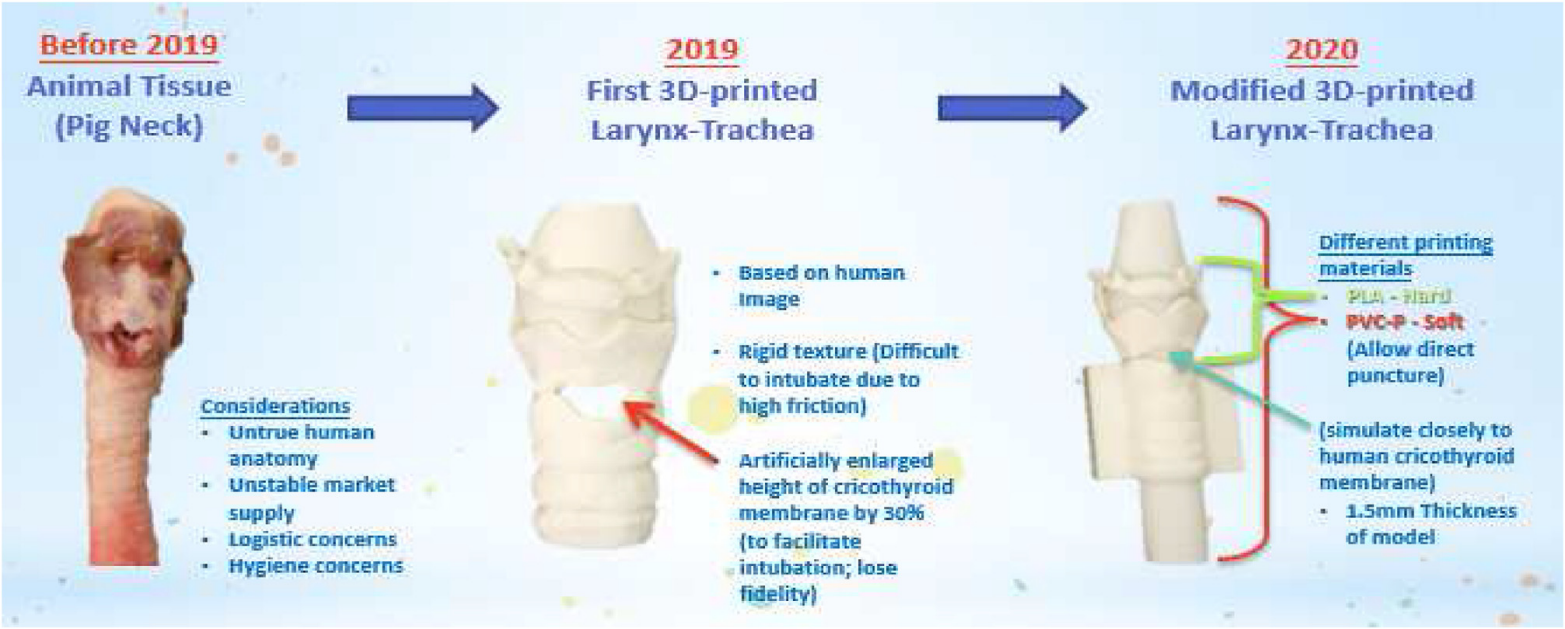
Comparison among core part of training models.

Since 2013, Cricothyroidotomy had been incorporated into a local “Advanced Surgical Trauma Course” where pig larynx-trachea models were used as a traditional training modality. The invasion of the COVID-19 pandemic accelerated the organizational needs of developing an alternative training curriculum and model. Due to untrue human anatomy, unstable market supply, and logistical and hygiene concerns, training director approached principal investigator in the organizing committee meeting for replacing animal model by self-invented simulator (Figure 1). The following paragraphs addressed some evidence of training effectiveness and build hypotheses based on Kirkpatrick models.

### 1.2 Literature on training effectiveness

#### 1.2.1 Live porcine model

Traditionally, porcine models served as a socially accepted way for Criothyroidotomy training. Stefanidis and colleagues conducted a Randomized Controlled Trial (RCT) to examine performance of junior medical staff in operation room after training, showing that those from experimental group with porcine model, compared with those from control group with didactic lecture only, had significant improvement in knowledge acquisition and skill transfer [17]. In a recent observational study, Deonarain and colleagues invited 7 otolaryngologists and head-and-neck surgeons to validate simulation training tool for open-airway surgery using live porcine model [18]. Over 90% agreed that simulator could enhance life-saving skills in applying tracheostomy following similar surgical airway procedures on throat effectively.

#### 1.2.2 Live porcine vs. innovative simulator

In a structural interview, Bukoski and colleagues identified that all 25 senior medics preferred live porcine to low-fidelity part-task trainer owing to optimal tactile sensation and physiologic responses [14]. Another research team led by Pandian conducted a RCT for 48 medical students on Cricothyroidotomy training with live porcine and high-fidelity surgical manikin [19]. Consistent with two studies piloted by the research teams of Hall and Savage [20, 21], their study showed insignificant difference in training effectiveness and in change of confidence between live porcine and manikin group.

Besides, no significant difference in task completion time was identified [19]. Thirty-five junior anesthesiologists who performed timed task after training with porcine model showed no additional benefits when compared with those trained with low-fidelity simulator [22].

Porcine model might weaken training effectiveness due to differences in anatomical structure and size from human cadaver [14, 19]. Despite human cadaver as alternative, their usage appears undesirable due to concerns about hygiene, market supply, and operation costs [23, 24].

#### 1.2.3 3D-printing and innovation

Usability is showcased when innovation meets application. Hughes and colleagues developed a Cricothyroidotomy simulator using 3D printing technology, silicone, and artificial blood pad [25]. Of 52 emergency trainees, 43 reflected on their experiences as satisfactory with the bleeding effect and skin texture, and 50% higher comfort level than training with porcine [25]. Similarly, Calvo and colleagues set an exemplar of proof-of-concept study, combining 3D printed trachea model, air pump, blood pad, and pork belly to enhance realism and human physiology at low costs [26]. With demonstration by 11 Intensive care trainees, airway surgical experts were satisfied with tactile fidelity, whereas “resistance or friction” on intubation process were high [26]. However, sample size of the study was small, without investigation on training effectiveness at higher level, in terms of learning effect and its transfer to clinical setting.

Urdiales and colleagues evenly distributed 90 doctors into intervention groups: (i) lecture, (ii) low-fidelity simulator, (iii) high-fidelity simulator [27]. Being examined by 20 multiple choice questions, groups with either simulator statistically outweighed lecture group, representing best performance in knowledge acquisition and retention in group with hands-on experience [27]. Since Cricothyroidotomy, as sort of hands-on surgical skills on psychomotor domain, could never ever be acquired by training without haptic and tactile sensation [28].

#### 1.2.4 Research gaps and study hypotheses

This is the first study applying Kirkpatrick model to assess training effectiveness of Cricothyroidotomy training in order to inform hospital management decision of continuation or discontinuation in training using new simulator and standard curriculum [16, 29]. To date, no existing academic paper has covered how innovative 3D printed simulator as replacement with standardized curriculum used to train up healthcare professionals’ knowledge and skills in surgical airway management [20, 24, 30, 31]. The study aims to evaluate training effectiveness of the new training curriculum and its organizational impacts using up to Level-3 (Behavior) of Kirkpatrick model. Based on research findings aforementioned, the authors postulated that:

• There were significant changes at Level-1 (Reaction) when comparing participants' satisfaction with new model than that with the old one (Hypothesis 1).
• There was no significant change at Level-2 (Learning) in time of completion when trained by new model (Hypothesis 2).
• There was no significant change at Level-3 (Behavior), such as confidence, when comparing scores of personal strengths using new simulator than that from average scores of past sessions using porcine model (Hypothesis 3).

## 2 Methods

### 2.1 Design

This is a hospital-based post-training evaluation under observational descriptive study design. Hospital Authority serves as the statutory body for all public hospitals and related clinics in the Hong Kong Special Administrative Region. Multi-Disciplinary Simulation and Skills Centre (hereinafter referred to as “the center”), governed by one of the public hospitals named Queen Elizabeth Hospital, has been providing healthcare professionals with ample high-fidelity simulation training opportunities. In collaboration with consultant surgeon, consultant anesthesiologist, consultant intensivist, center manager, biomedical engineering technicians, and research officer specialized in occupational psychology and biostatistics, a professional workgroup was established to carry out the study to: (i) implement new training curriculum for Cricothyroidotomy training (with newly developed simulator using 3D printing technology and standardized workflow) and, (ii) evaluate training effectiveness following the organization change in the hospital (For technical part in details, see Appendix 1; For standardized training curriculum, see Appendix 2).

### 2.2 Procedures

#### 2.2.1 Participants

Taking into account training needs, training capacities, as well as availability of manpower under protected hours for training, 70 medical staff and 10 nursing staff were recruited from clinical departments in the Hospital Authority within the time frame between January 2020 and March 2021. In trauma team management, emergency physicians and general surgeons have higher probability (or in other words, training needs and priority) to perform Cricothyroidotomy [32]. Other key members in trauma team, including orthopedic surgeons, neurosurgeons, anesthesiologists, intensivists, and emergency nurses were required to fulfil training requirement for this life-saving procedure, regardless of their likelihood of performing Cricothyroidotomy procedures on real patients. Agreed by the steering committee board for effective resource allocation, 50 and 30 training quotas were assigned to the 1st tier and the 2nd tier recruitment, respectively.

#### 2.2.2 Recruitment

Random sampling would not be feasible because training quotas were scarce and must be allocated to potential participants with training needs. For reasons of perceived fairness in selection of participants and minimization of training needs and service gap, all participants were nominated from heads of department of Surgery, Accident and Emergency, Orthopedics, Neurosurgery, Anesthesiology, and Intensive Care Unit in five trauma centers under the Hospital Authority. On fixed-quota nomination basis, there were no concerns about low participation rates.

#### 2.2.3 Inclusion Criteria

i) Any medical or nursing staff involved in trauma management under the Hospital Authority with fixed quota:
• 1^st^ Tier: 50 staff, including 35 general surgeons, and 15 emergency physicians
• 2^nd^ Tier: 30 staff, including 6 neurosurgeons, 4 orthopedic surgeons, 10 anesthesiologist or intensivists, and 10 emergency nurses
ii) Pre-requisite in the Advanced Trauma Life Support training to ensure acquisition of foundation of life-saving knowledge and skills for all stated frontline professionals including emergency nurses

#### 2.2.4 Exclusion Criteria

i) Age 18 or below
ii) Unable to give informed consent
iii) Unable to complete training
iv) Unable to complete questionnaires

#### 2.2.5 Ethical considerations & procedures

Organization agreement and official ethical approval from the Ethics Committee of the University of Leicester have been granted prior to data collection (UoL Ethics Reference no.: 29837). Aligned with standard operation procedures of the center, all participants who would like to join in the simulation training were required to complete written informed consent on confidentiality issues and permission for use of data by the center through electronic registration system in advance. Due to COVID-19 pandemic, infection control measures suggested by the infection control team were applied to check participants' temperature using touchless forehead infrared thermometer and report on health declaration on arrival of participants. In-class behavior, including removal of surgical mask, consumption of any food or beverage, and social distancing less than 1.5 m were strictly prohibited. Regarding measures to protect privacy of participants, all electronic research data were managed in line with hospital policy in handling, storage, and destruction of patients’ medical record, even no patient records were involved throughout the study. All data were encrypted and saved in a desktop computer solely accessible by the authors, and the raw data would be destroyed two years after completion of the study. With specific coding of participants, personal identifiers could no longer be found in the anonymized dataset, with no chance of disclosure in data analysis.

### 2.3 Measurements – Simulation Training Course Evaluation (STCE)

#### 2.3.1 Contents

Complied with professional standards on “Education and Teaching” of Society for Simulation in Healthcare, all participants of simulation training were encouraged to complete a standardized course evaluation form using their smartphone or electronic device at the end of the training. The form named “Simulation Training Course Evaluation” (STCE) was made up of 35 items to evaluate post-training effect. All rating items were measured on 5-point Likert scales. Calculating average score of items could generate certain sub-scores under two categories, namely “simulation training” and “personal strengths” adopted from Cheung and colleagues under the leadership of Chia [33, 34, 35].

“Simulation training” has 6 sub-components for Kirkpatrick's Level-1 (Reaction), including:

1. Training needs: Training objectives for service quality and safety
2. Training design: Enablers of successful implementation of simulation training
3. Simulation: Experiences of Cricothyroidotomy through mimicking the procedure in controlled environment
4. Debriefing: Evoking insights of participants by reviewing and reflecting on the procedures
5. Instructor feedback: Professional advice for further enhancement
6. Satisfaction: Perceived positive experience in healthcare simulation training

“Personal strengths” have 5 sub-components for Kirkpatrick's Level-3 (Behavior), including:

1. Assertiveness: Speak-up for potential risk procedure
2. Mental preparedness: Mental capacity for handling emergency condition
3. Self-efficacy: Sense of mastery in executing Cricothyroidotomy
4. Internal locus of control: Sense of control of patients' lives with practical skills of Cricothyroidotomy
5. Internal locus of responsibility: Sense of morality and accountability to patient lives

#### 2.3.2 Validation

The questionnaire was reviewed by the steering committee and high management on bi-annual basis to ensure its applicability to address simulation training effectiveness. Following validation in 2018 and latest revision in May 2020, the questionnaire was updated with sentence simplification, question reconstruction, and additional items to further understand how psychological aspects (personal strengths components mentioned) influence overall satisfaction, knowledge and skills acquisition, as well as transfer to operation [33]. This questionnaire showed excellent inter-item consistency on components related to simulation training (*Cronbach's Alpha* = .92) and personal strengths (Cronbach's alpha = .96). Content validity of entire questionnaire was high as well (*S-CVI/Ave* = .96, *S-CVI/UA* = .87) [34, 35].

### 2.4 Data analysis

#### 2.4.1 Statistical methods

Descriptive Statistics on demographic information, including type of professional/specialty, gender, training priority, seniority, and prior training experience with procine model, were reported by counts and percentages (Table 1).

**Table 1:** Demographic characteristics of participants in cricothyroidotomy training.

Character (N = 80)		Counts	(%)
Discipline	*General Surgeon*	35	43.8
	*Emergency Physician*	15	18.7
	*Emergency Nurse*	10	12.5
	*Neurosurgeon*	6	7.5
	*Anesthesiologist/Intensivist*	10	12.5
	*Orthopedic Surgeon*	4	5
Priority	*First Tier*	50	62.5
	*Second Tier*	30	37.5
Gender	*Female*	38	47.5
	*Male*	42	52.5
Seniority	*1–5*	40	50
	*6–10*	26	32.5
	*11–15*	8	10
	*>16*	6	7.5
Training Experience	*New Training Program only*	35	43.7
	*Both New Training Program and Traditional Porcine Model*	45	56.3

In order to provide quantifiable measurements for inferential analysis, all items were set with responses using 5-point Likert scales (1 = Strongly Disagree; 3 = Neutral; 5 = Strongly Satisfied; 4 or above out of 5 would be considered as “satisfactory”) as opposed to nominal or dichotomous answer. Primary investigation was to compare parameters of simulation training and personal strengths using the new training curriculum and simulator with that using unstandardized curriculum and traditional porcine model by one-sample t-test using SPSS software (ver. 25, IBM Corp.). Owing to varied composition of participants, training curriculum and use of revised questionnaire (with slight modifications on statement wordings for enhanced clarity), a rough reference point for all parameters was set at 3.8 out of 5 based on average scores from past training records before 2020.

## 3 Results

### 3.1 Level-1 (reaction)

Regarding reaction-related results in STCE, sub-categories including (i) Training needs, (ii) Training design; (iii) Simulation, (iv) Debriefing, (v) Instructor feedback, and (vi) Satisfaction were evaluated. Overall scores of 6 subcategory ranged from 4.39 to 4.44 out of 5, which were about +0.6 points away from reference point of previous training using old curriculum (see Table 2). Using one-sample t-tests, statistically significant differences were found between participant scores in current session and reference point with old curriculum for all stated sub-categories, *t(79)* = 4.63 to 4.85, *p* < 0.1.

**Table 2:** Comparing participant scoring on all domain after training with new curriculum with that with old curriculum.

	All participants (N = 80) M ± SD	∗ Difference from Reference Point	One-sample T-test P-value
Simulation Training Domains			
*Training needs*	4.39 ± .54	+0.59	.001
*Training design*	4.43 ± .52	+0.63	.000
*Simulation*	4.42 ± .59	+0.62	.000
*Debriefing*	4.39 ± .53	+0.59	.001
*Instructor feedback*	4.42 ± .53	+0.62	.000
*Satisfaction*	4.44 ± .55	+0.64	.000
Personal Strength Domains			
*Assertiveness*	4.41 ± .54	+0.61	.000
*Mental preparedness*	4.39 ± .58	+0.59	.001
*Self-efficacy*	4.43 ± .61	+0.63	.000
*Internal locus of control*	4.46 ± .62	+0.66	.000
*Internal locus of responsibility*	4.45 ± .59	+0.65	.000

Note. N = Valid number of participants; M = Mean; SD = Standard Deviation

∗3.8 out of 5 as the reference point on Simulation Training Course Evaluation in previous Advanced Surgical Trauma Course; No reference point for Scales of Emergency Surgical Airway Simulator.

### 3.2 Level-2 (learning)

Acquisition of Cricothyroidotomy skills was assessed by simulation instructor with standardized procedures (see Appendix 3). Full compliance of all necessary procedures without errors (in terms of sequence and puncture site) within 2 min would result in “Pass”. All participants (*N* = 80) passed this summative assessment on Cricothyroidotomy skills after mass demonstration and one-off practice (*Successful Rate* = 100%) compared with that in 2018 and 2019 at about 75%.

### 3.3 Level-3 (behavior)

Refer to STCE, items about (i) Assertiveness, (ii) Mental preparedness, (iii) Self-efficacy, (iv) Internal locus of control, (v) Internal locus of responsibility were evaluated as behavior-related scoring. Overall scores of 6 subcategory ranged from 4.39 to 4.44 out of 5, which were about +0.6 points away from reference point of previous training using old curriculum (see Table 2). Using one-sample t-tests, statistically significant differences were found between participant scores in current session and reference point with old curriculum for all stated sub-categories, *t(79)* = 4.63 to 4.85, *p* < .01.

## 4 Discussion

Overall training effectiveness of Cricothyroidotomy sessions was excellent. Under Kirkpatrick model, simulation training in Cricothyroidotomy procedure using new curriculum and simulators has been proven to be useful for healthcare professionals involved in trauma service management.

### 4.1 Level-1 (reaction)

Hypothesis 1 was supported by the result. There were significant changes at Level-1 (Reaction) when comparing participants' satisfaction with new model than that with the old one. All participants rated 4 (Agree) or 5 (Strongly Agree) on all items of 5-point Likert scales in STCE, showing their high satisfaction with overall arrangement for immersive learning experience before (Familiarization/Briefing), during (Simulation with instructors’ feedback), and after training (Debriefing for consolidation of knowledge).

### 4.2 Level-2 (learning)

Hypothesis 2 was supported by the result that no significant change at Level-2 (Learning) in time of completion when trained by new model. To ensure skill attainment, expert instructor and technician observed participants’ performance and found that all of them reached proficiency of 4-step life-saving procedure individually and completed within time limits. Even though the time of completion in current study is similar to that in the past, the successful rate of participants has raised by 33% which achieved 100% successful rate at the end.

### 4.3 Level-3 (behavior)

Hypothesis 3 was not supported by the result. There was significant change of most personal strengths parameters (including self-efficacy, a similar attribute as confidence), when comparing that with average scores of past sessions using porcine model. Positive responses were extremely high on domains of personal strengths in STCE. Beyond achieving the primary objective of this skill-based training for acquisition of practical skills in Cricothyroidotomy, the findings provided a concrete evidence that structured training coupled with the new training technology and standardized curriculum could optimize translational effect on communication skills or assertiveness in work setting [36].

### 4.4 Review on preference of simulator and training curriculum

#### 4.4.1 Preference of simulator

Previous studies suggested that self-efficacy and resilience would be gained from training modality with live tissues [14, 26]. Without contradictions, this study showed that simulator without animal tissues could be fostering personal strengths (i.e. mental preparedness, self-efficacy, internal locus of control and responsibility) as well. It is speculated that participants with prior experience in porcine model might perceive higher physical and functional fidelity using new model which implies its excellent features of “bleeding, skin texture” in psychologically safe environment without hygiene concerns [25]. Consistent with previous results, no differences were found in completion time between participants with or without prior training experience in procinemodel [22]. Despite small association between realism and training effects, the group with such prior experience showed lower training effectiveness [14, 19]. Irrespective of prior experience in surgical airway training, the study supported synthesized perspective from systematic reviews that medical education could help medical trainees foster leadership, such as confidence, in trauma management setting [37, 38].

#### 4.4.2 Training curriculum and tool

This study reaffirmed that training needs would be considered as the most important factor in planning and review of simulation training. Satisfactory training curriculum and simulation procedures with international standards [39], timely feedback and effective debriefing skills of instructor are facilitators for training effectiveness and quality assurance. Besides, personal strengths would be more likely to foster when training objectives were met [14, 26]. All satisfactory findings reflected service needs perceived by high management and steering committee members of the Advanced Surgical Trauma Course were correspondent with training needs of medical and nursing staff in their position for trauma management [9, 11, 12, 13, 40].

Insights into the perception of usability were identified. Efficacy in use of simulator would be higher when the simulator performed well in mimicking entire Cricothyroidotomy procedure in an innovative approach under training environment with physical and psychological safety (e.g., physical comfort, hygiene) [26, 27]. The more the vibes of safety were secured in training, the more likely participants would show acceptance to use new simulator instead of procine model in knowledge and skills acquisition and its transfer to daily operation [16, 25].

#### 4.4.3 Pearls and pitfalls

This is the first evaluation on training effectiveness of Cricothyroidotomy using new curriculum and simulator under Kirkpatrick model, showing feature of research initiatives and applicability of innovations as an opportunity yet by the same token facing inadequate support of literature for comparison of research findings as a risk. Although applying Kirkpatrick model to evaluate simulation training effectiveness could fill part of knowledge gaps, several limitations have been identified:

#### 4.4.4 Sampling issues

With limitation of costs and feasibility matters (lacking in supply of pig and out of service for animal lab) amid the COVID-19 pandemic, no trials could be done for training with two arms of utilizing pigs and 3D printed alternative. No randomization or random sampling method was applied as all participants were nomination from heads of departments or their delegates to fill the training quotas. Would there be any threats of external validity? Since most related studies were from heterogeneous sample and with high variation in methodology (e.g., risk of bias, imprecise process and/or measurement… etc), comparative conclusion drawn from results without taken into account sample sources could be misleading [[39], [41], [42]]. For instance, doctors who preferred simulation with live tissues were recruited from military, not from mainstream medical school nor from general hospital setting [14]. Interestingly, military doctors might be highly motivated by (or inversely less sensitive to) live blood/skin texture, not by anatomical structure/procedural in Cricothyroidotomy [20].

#### 4.4.5 Response bias

Response bias from self-report measurements could not be ruled out in this study. To be specific, acquiescence (not processing true meaning when answering questions), social desirability (faking good), and maligning (faking bad) may happen [43]. The authors have explained to all participants the value of the study as to facilitating hospital management decision based on their genuine responses. Although self-report responses might be subjective and unable to verify, anonymity measures and setting with privacy should have mitigated the tendency of not giving true answers.

#### 4.4.6 Restriction in data analysis

This study compared training satisfaction and personal strengths from current session with that from reference point of past training records before 2020. The authors decided to use reference score instead of exact score for data comparison because the STCE used before the commencement of this study in 2020 was not yet revised and validated by subject matter experts in simulation training and psychometrics. Another issue was level of measurements. In order to enhance response rates for demographic items participants preferred not to say, “years of seniority” for instance, range of years were provided as opposed to free entry which requires a specific number.

### 4.5 Future direction of cricothyroidotomy research

Depending on study purpose (e.g., review or audit on existing prioritization policy of training quota), future study investigating between-group effects following implementation of new surgical airway training curriculum and simulator would inform decision of high management for effective resources allocation. Research excellence may contribute in both conceptual and practical ways. For example, should limited training quotas be prioritized for emergency physician or surgical trainees with higher seniority in specialty or shared equally with all major stakeholder in trauma team? Except effect of resource allocation, applicability of innovative training modality for psychomotor skills development using virtual reality may be explored [28, 44, 45, 46, 47, 48], especially in times of metaverse where social distancing and medical education could be largely maintained via virtual learning environment during post-pandemic phase.

### 4.6 Conclusion

The findings suggested that application of a state-of-the-art training tools to advanced surgical skills training could yield positive change in satisfaction of participants, knowledge and skills acquisition, and personal strengths unequivocally transferable to trauma management setting.

## Declarations

### Author contribution statement

Dr. N.-H. Chia, Victor K.-L. Cheung, Mr. Taurus K.-Y. Wong, Ms. Madeleine L.-Y. Lam, Ms. Iris W.-K. Cheung, Ms. S.-S. So.

Dr. Eric H.-K. So, Dr. George W.-Y. Ng: Conceived and designed the experiments; Performed the experiments; Analyzed and interpreted the data; Contributed reagents, materials, analysis tools or data; Wrote the paper.

### Funding statement

This research did not receive any specific grant from funding agencies in the public, commercial, or not-for-profit sectors.

### Data availability statement

Data will be made available on request.

### Declaration of interest’s statement

The authors declare the following conflict of interests: Some of the authors are owners of Hong Kong Patent (#30041806) for “Emergency Surgical Airway Surgical Simulation Device”.

### Additional information

No additional information is available for this paper.
