# Supplementary material for: Harnessing power of simulation training effectiveness with Kirkpatrick model in emergency surgical airway procedures
Source: Heliyon. 2022 Oct 6;8(10):e10886. doi: 10.1016/j.heliyon.2022.e10886 (PMC9576887; doi:10.1016/j.heliyon.2022.e10886)
Supplement: Appendix [file mmc1.docx]

**APPENDIXES**

#

# **Appendix 1. Setup of Emergency Surgical Airway Simulator (ESAS)**


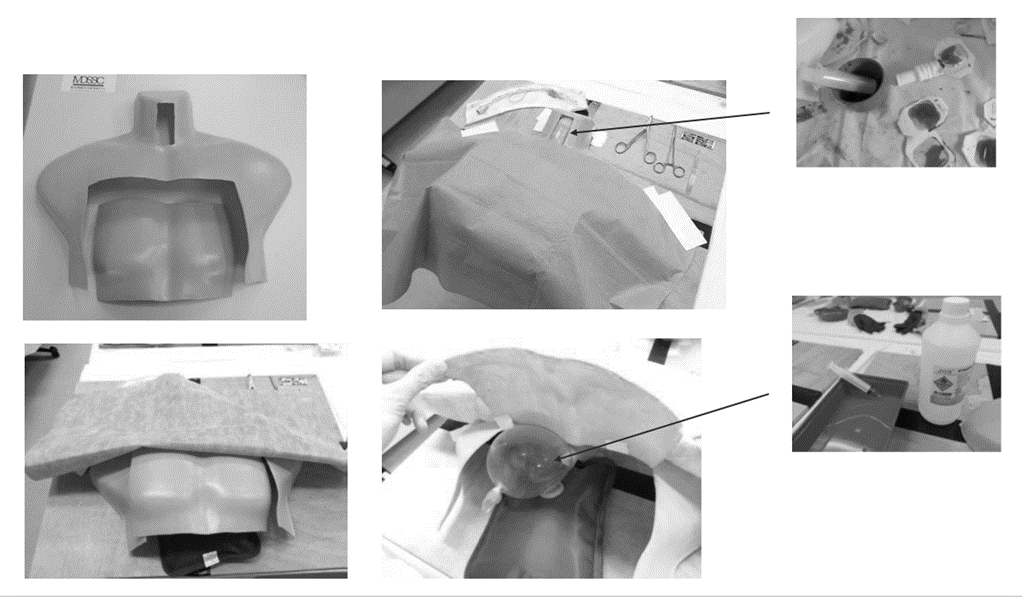


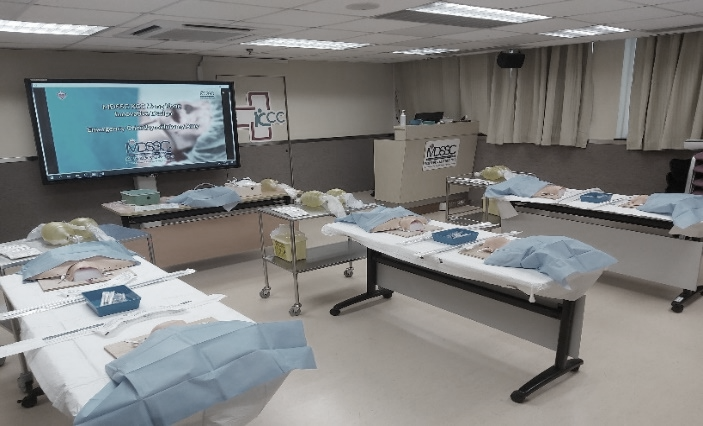

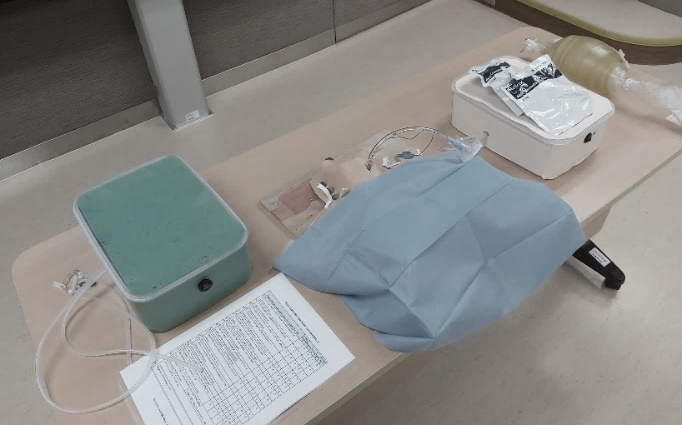


**Level of fidelity with Samples**

- **Low fidelity:** use real “pig neck” or low technology model dissimilar to human anatomical structure or ratio
- **Medium fidelity:** use basic “3D printed larynx-trachea model” with 1:1 human anatomical structure (for deliberate practice on skills with tactile sensation); or virtual reality program (for strengthening memory of surgical procedure)
- **High fidelity:** Simulator (or part-task trainer) combining 3D printed technology with other innovative approach to simulate not only the shape of the anatomical structure for surgery but also the observable effect following the simulated surgical procedure (e.g., see “red water” coming out when cutting on simulated skin, hear sound of air leaks when cutting on cricothyroid membrane… etc)
- **Full-motion fidelity:** beyond individual skills training, usually in teams and on either mixed mode (using 2 or above training modalities) or hybrid mode (combining simulators with manikin or simulated patients during the scenario)

### **Appendix 2. Standardized Training Curriculum of Cricothyroidotomy**

### Professional Standards

This program was designed in compliance with international standards of emergency airway management:

1. Canadian Airway Focus Group (CAFG) guidelines 2013, suggested that all members involved in trauma management should be trained their skills of Cricothyroidotomy [1].
2. Difficult Airway Society (DAS) 2015 guidelines for “Technique for scalpel Cricothyroidotomy” emphasized importance of both technical skills and skills concerning human factors (e.g., teamwork and leadership, communication, situation awareness) for reinforcing and retraining skills [2].

#### Training Objectives

Participants were able to i) understand the situation of cannot-intubate-cannot-oxygenate (CICO) and standard emergency surgical airway procedure (Cricothyroidotomy) with international guideline and ii) perform bougie-assisted surgical Cricothyroidotomy and maintain ventilation for patients within 2 minutes.

#### Procedures of Cricothyroidotomy Training

During registration process, participants were provided with information letter and consent form under assistance of research officer. After reading them thoroughly without further questions, they would give e-signature on digital consent form under witness of designated staff. Participants underwent the training according to the standard rundown.

| **Rundown of Procedural Simulation Training for Cricothyroidotomy** | |
| --- | --- |
| **Flow** | **Content/ Remarks** |
| 1. Briefing and Familiarization | - Training objectives - Familiarization of training environment (e.g., simulator, equipment) |
| 1. Introduction | 1. Cannot Intubate Cannot Oxygenate (CICO) condition 2. Anatomical physiology of human neck 3. International standard: 2013 Canadian Airway Focus Group (CAFG) and 2015 Difficult Airway Society (DAS) [1-2] |
| 1. Live Demonstration | with verbal commentary of standard procedure of bougie-assisted Cricothyroidotomy |
| 1. One-off Practice and Skill Assessment of Cricothyroidotomy | within 2 minutes under observation by instructor, one-by-one   - Step 1: Midline longitudinal incision over skin - Step 2: Transverse incision through the membrane - Step 3: Endotracheal tube insertion with or without a bougie - Step 4: Bagging after connecting Ambu-bag with endotracheal tube |
| 1. Debriefing | Review simulated procedure with instructor |
| 1. Wrap-up | Recap with take-away message |
| 1. Evaluation | Completion of training evaluation via QR code scanning |
| [1] Law JA, Broemling N, Cooper RM, et al. The difficult airway with recommendations for management--part 2--the anticipated difficult airway. *Can J Anaesth.* 2013;60(11):1119-1138. [2] Frerk C, Mitchell VS, McNarry AF, et al. Difficult Airway Society 2015 guidelines for management of unanticipated difficult intubation in adults. *Br J Anaesth.* 2015;115(6):827-848. | |

### **Appendix 3. Visualization of 4-Step Procedure of Cricothyroidotomy and Checking**
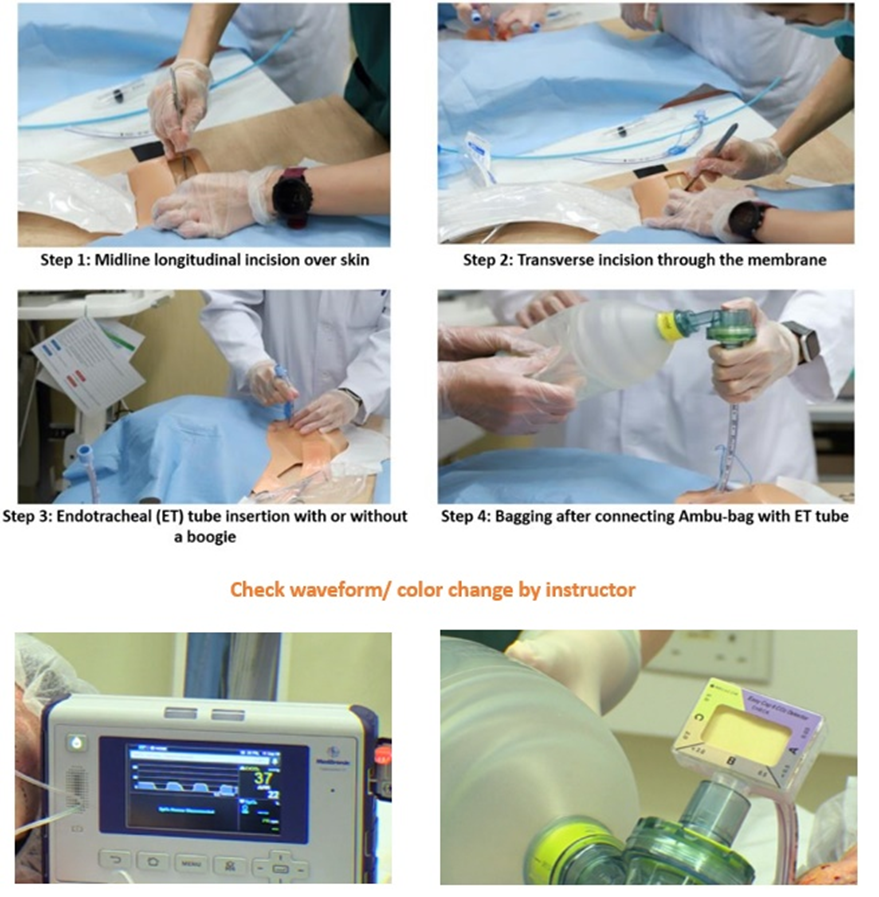


**Endpoint**

Display of either i) waveform in square shape on monitor or ii) temporary switch between yellow (mimicking expiration) and purple (mimicking inspiration) color on colorimetric capnometer during manual bagging indicates “mission complete”.
